# The Introductory Addresses at the Medical Schools

**Published:** 1896-10-10

**Authors:** 


### The Introductory Addresses at the Medical Schools.

"From grave to gay; from lively to severe;" the opening addresses at the various London and provincial medical schools last week presented almost every variety of speculation, instruction, and even entertainment which the most exacting medical student could possibly wish for. Medical men, it is well known, are impatient of much speech. Deeds they prefer to words; and not very long ago it was seriously contemplated to extinguish the opening address at the commencement of the medical session. That was not done, we are glad to remember; and now that the leading metropolitan newspapers are reproducing many of the opening addresses almost in full, there is little fear that it will be done.

At the Middlesex Hospital, Dr. Essex Wynter took quite the tone of the experienced, travelled, and widely-read man of the world; and endeavoured to impart to the assembled students some of his own enthusiasm for philosophic thinking, and for the employment of those methods of study which lead to a high mastery of the principles and details of the medical art. The medical student, Dr. Wynter thinks, is obliged to learn so very much that, with the short time at his disposal, he can learn nothing well. When, however, he has succeeded in "satisfying all his examiners" he should set about some independent study, should begin to sift wheat from chaff, should seek hospital appointments, should travel abroad to foreign medical schools; and generally should make an intelligent and prolonged effort to become a master in his art. One point Dr. Wynter insisted upon, which, though neither medical nor scientific, is really of very great importance indeed to medical men; and most of all, perhaps, to general practitioners. "It is a widely-accepted statement," said he, "that doctors as a body are bad men of business." "To that," he continued, "more than to lack of ability in professional matters, could be traced want of success in many instances." Will young doctors lay this to heart? We fear not. But if they do not we are quite sure that many of them will be compelled to take it to heart at a later stage of their career; and when perhaps all the "taking to heart" in the world will not save them from the bitterness of professional failure, and even ruin. Such an address as Dr. Wynter's could not but be of great service both to "freshmen" and advanced students; and it would have been a great pity if it had not been delivered.

At University College, Dr. Sidney Martin occupied quite different ground. But his address, severely scientific and intensely practical, was of a nature to open the eyes of the assembled students to the greatness as well as to the difficulty of the subjects they were called upon to study, and to make them then and there resolve either to "quit themselves like men" in the days of their pupilage, or to give up the too arduous study of medicine at once and for ever. Dr. Sidney Martin is a capable thinker as well as a learned physician. One would fain hope that most of his youthful listeners took pains both to comprehend his intellectual position and to share in his enthusiasm for medicine as a study and a life-work. Medicine, he said, "has this great advantage—it possesses both a scientific and a practical side." Now, what does this mean to the true student of medicine? It means that he has chosen a calling which will keep him fresh and young in mind to the very last day of his life. The "scientific side" is a side which can never grow old, never pall upon the intellectual appetite, never fail to stimulate, to quicken, and to rejuvenate mental activity so long as mental and physical activity can be made to endure. The "practical side" is the true complement of the "scientific" in medicine; and the student who is "human" as well as devoted to learning finds in it the utmost satisfaction of all his moral and sympathetic aspirations and enthusiasms. Medicine, indeed, when rightly understood, is second to no calling in its intellectual devotion and its noble humanity. Happy are those students who see it in this, its only true, light.

At St. George's, Mr. Adams Frost was sarcastic at the expense of the dullards, and maintained that, notwithstanding the entrance examination, a large number of men became medical students who were quite unfit to become medical men. He did not think very highly of the qualifications required to pass examinations; nevertheless, they did serve to keep out of the profession certain stupid people who, however hard they worked, never seemed to learn anything; who, having shown no capacity for learning when they were boys, were not likely to develop it as men, and having been dunces at school would be dunces all their lives.

At St. Thomas's, however, Lord Justice Lindley held out hope even for the backward. Much as he congratulated the prize winners, the unsuccessful competitors should not despair, he said, for men sometimes developed late in life, and one of the greatest lawyers of the present generation, the late Lord Selborne, was plucked in "Mods" at his University.

At St. Mary's, Mr. Morton Smale descanted eloquently on the evils of patent medicines, and protested against the assumption made by some writers that the advance of education enabled people to treat minor ailments for themselves; while at Westminster, Dr. Wills dived deep into literature, and from the novels of various periods showed how much better was the social position now occupied by medical men than it was in days gone by.

At the London School of Medicine for Women, a man, Mr. Bryce Barron, F.R.C.S., was selected to give the address, which seemed rather a pity. If a woman can do anything supremely well, it is talking; and it would have been decidedly interesting to have had an original lecture from one of the very clever medical ladies who haunt Gray's Inn Road, for purposes of comparison with the duller addresses of her male contemporaries.

The opening addresses delivered at the medical schools have been almost without exception of a character not merely to justify the "wide" circulation which they have obtained in the daily press, but also to add to the reputation of the profession as a whole for general and special culture and high intellectual capacity. The great world has never thought too highly of us. The reason for that has been, at any rate during the past half-century, that it has not known us well. The time is apparently coming when it will know us better; and if that should prove to be so, the great world itself will gain a good deal, and we, on our part, shall certainly not lose.

